# Food sensitisation profiles in school-aged children from China and Russia

**DOI:** 10.1186/2045-7022-5-S3-P42

**Published:** 2015-03-30

**Authors:** Mareen Datema, Serge Versteeg, Edmund Yung, Olga Fedorova, James Potts, Ischa Kummeling, Montserrat Fernández-Rivas, Ludmilla Ogorodova, Peter Burney, Maria Yazdanbakhsh, Anand Mahesh Padukudru, Gary Wong, Ronald Van Ree

**Affiliations:** 1 Academic Medical Centre, Amsterdam, The Netherlands; 2 Chinese University of Hong Kong, Hong Kong, China; 3 Siberian State Medical University, Tomsk, Russia; 4 National Heart and Lung Institute, Imperial College, London, United Kingdom; 5 Hospital Clinico San Carlos, IdISSC, Madrid, Spain; 6 Leiden University Medical Centre, Leiden, The Netherlands; 7 Allergy Asthma Associates, Mysore, India

## Background

Compared to Europe, little is known about sensitization to food and inhalant sources in Russia and China.

## Objective

To explore sensitization profiles of school-aged children in urban and rural settings from Russia (Western Siberia) and South-Eastern China.

## Methods

In the EuroPrevall-INCO survey, primary school children (7-10 years) from randomly selected schools in China (Hong Kong [urban/n=401], Guangzhou [urban/n=402], Shaoguan [rural/n=349]) and Russia (Tomsk [urban&rural/n=1230]) were first screened by a short questionnaire for self-reported food allergy. Subsequently, cases and randomly sampled controls underwent a more detailed clinical evaluation by standardized questionnaire, and all were assessed for sensitization by SPT and ImmunoCAP analysis against 27 different foods and 8 inhalants. A subset from Hong Kong (n=216) and Tomsk (n=318) was further screened for sensitization to 56 food and 6 inhalant allergens by custom-made microarray.

## Results

Shrimp (7-25%) and crab (4-9%) were the most common food sensitizations in Chinese children, except in Hong Kong, where egg (17%) and milk (15%) sensitization was higher than that to shrimp (13%). In all other sites <7% was sensitized to milk and egg. Children from Tomsk were mostly sensitized to hazelnuts and fruits (~6%) and this was associated with birch pollen and Bet v 1 sensitization. Hazelnut sensitization was <4% in China and fruit sensitization was almost absent in Guangzhou. Sensitization to house dust mite (HDM) was exceptionally high in China (p39%), especially in Hong Kong (73%), compared to Tomsk (13%). IgE titers to HDM were significantly lower in Shaoguan (median 2.0 kUA/L) than in Hong Kong and Guangzhou (median 71.8 and 26.2 kUA/L, respectively).

## Conclusion

Sensitization profiles in Tomsk are dominated by birch pollen cross-reactivity, similar to what is common in Central and Northern Europe. In contrast, sensitization to HDM and sea foods dominated the picture in China. Whether house dust mite sensitization is (partly) at the basis of the high prevalence of seafood sensitization (e.g. via Der p 10/tropomyosin) or whether it is the seafood-rich diet, is currently under investigation.

